# Flash Thermography to Evaluate Porosity in Carbon Fiber Reinforced Polymer (CFRPs)

**DOI:** 10.3390/ma7031483

**Published:** 2014-02-26

**Authors:** Carosena Meola, Cinzia Toscano

**Affiliations:** 1 Department of Industrial Engineering—Aerospace Division, University of Naples Federico II, Via Claudio 21, 80125 Napoli, Italy; 2 C.I.R.A.—Italian Aerospace Research Centre, via Maiorise sn, 81043 Capua (CE), Italy; E-Mail: c.toscano@cira.it

**Keywords:** composites, porosity evaluation, flash thermography

## Abstract

It is a fact that the presence of porosity in composites has detrimental effects on their mechanical properties. Then, due to the high probability of void formation during manufacturing processes, it is necessary to have the availability of non-destructive evaluation techniques, which may be able to discover the presence and the distribution of porosity in the final parts. In recent years, flash thermography has emerged as the most valuable method, but it is still not adequately enclosed in the industrial enterprise. The main reason of this is the lack of sufficient quantitative data for a full validation of such a technique. The intention of the present work is to supply an overview on the current state-of-the-art regarding the use of flash thermography to evaluate the porosity percentage in fiber reinforced composite materials and to present the latest results, which are gathered by the authors, on porous carbon fiber reinforced polymer laminates. To this end, several coupons of two different stacking sequences and including a different amount of porosity are fabricated and inspected with both non-destructive and destructive testing techniques. Data coming from non-destructive testing with either flash thermography or ultrasonics are plotted against the porosity percentage, which was previously estimated with the volumetric method. The new obtained results are a witness to the efficacy of flash thermography. Some key points that need further consideration are also highlighted.

## 1. Introduction

Composite materials are nowadays ever more massively employed in advanced engineering structures with their use in the transport industry, in civil infrastructures, in chemical equipment, *etc.* This success is justified by the many advantages they offer, amongst others, the possibility to choose both the raw materials and the manufacturing process to obtain a final product of desired characteristics. In civil aviation, composites are highly appreciated especially for their favorable stiffness-to-weight ratio, which involves a significant reduction in fuel burnt per seat per mile with a consequent significant decrease in the emission of CO_2_.

Carbon fiber reinforced polymers (CFRPs) are mostly employed in the construction of aircraft [1]. Within the mostly used hand lay-up manufacturing method, plies of fibers impregnated with resin (generally named prepregs) are overlaid, following a fixed stacking sequence, and cured in autoclave. The autoclave cycle involves the combined effects of temperature and pressure. Temperature is needed to activate and to control the chemical reactions in the resin, while pressure is used to squeeze off the excess resin, to consolidate the stacked plies and to minimize the amount of entrapped gas between the plies and within the resin [2]. Therefore, the action of temperature and pressure, as well as the cycle length are the main parameters to care for in order to assure the effectiveness of the curing process and, consequently, the overall quality of the final part.

Generally, the best curing temperature value is found through specific thermo-physical tests aimed at monitoring the dependence of the resin viscosity on the temperature and through the measure of the glass transition temperature. The determination of the optimum vacuum pressure value with its application time represents a difficult task to deal with [3]. Indeed, if both pressure value and application time are not optimized, voids could rise in the laminate [2]. As a main effect, the presence of voids reduces the interlaminar shear strength causing delamination (inter-lamina debonding) [4]. Besides, the presence of regions of fibers unsupported by the matrix can induce local stress concentration, with consequent severe degradation of strength and stiffness in-service. That is why the porosity level and its distribution within the whole final part must be known and taken into account.

However, the presence of a certain amount of voids is unavoidable [4]; it is important to know the level of voids that is acceptable in the specific application field. In fact, in many composite applications, the void content is quite critical, and levels above about 1% are not tolerable, such as in advanced composite dynamic aerospace structures, while in other applications, levels of 5% and higher can be tolerated [4]. Of course, lower void content means higher production costs, whereas loosening the quality control standards translates to a lower cost of the end products. Often, establishing the acceptable level of voids is a critical issue in designing composite structures, and then, the use of effective evaluation techniques is compulsory.

The intention of this paper is to present an overview of some of the most used methods, with particular attention devoted to the state-of-the-art on the use of flash thermography, which actually seems to be the most effective and advantageous method to assess the porosity percentage in composites. In particular, the overview involves data available in the literature integrated with the latest results, which were gathered by the authors, on porous CFRP laminates. At last, some key points that need further consideration are highlighted.

## 2. Results and Discussion

Basically, the porosity percentage can be determined through two approaches: destructive and non-destructive. Of course, destructive methods involve small proper samples and supply information only by statistical inference. On the contrary, the use of non-destructive techniques allows for the inspection of the whole part.

### 2.1. Destructive Methods

The porosity percentage can be estimated through three methods: volumetric, microscopy and acid digestion.

Measurements with the volumetric method are carried out by considering small samples, which may be either cut from a large laminate or prepared ad hoc, to be representative of a production lot. Basically, the method supplies measurements of density through weight in air and in water; then, the resulting porosity is calculated from the law of mixtures by knowing the average density [5]. In more details, each small piece, of known volume, is weighted (by a precision digital balance) to allow the calculation of the density, ρ, through the Archimedes’ principle. The law of mixtures gives:

$$\rho =\frac{\rho_{f}V_{f}+\rho_{r}V_{r}}{V_{f}+V_{r}}$$ (1)

where *V_f_* and *V_r_* respectively represent the volume of fibers and of resin; whereas ρ*_f_* and ρ*_r_*, are the density of the fibers and of the resin (ρ*_f_* and ρ*_r_* are provided by the prepreg lamina supplier). Equation (1) may be rewritten by introducing the volume of voids, *V_v_*:

$$\rho =\frac{\rho_{f}V_{f}+\rho_{r}V_{r}}{V_{f}+V_{r}+V_{v}}$$ (2)

Being *V_f_* + *V_r_* + *V_v_* = *V_T_* the actual total volume of the piece, from Equations (1) and (2), it is easy to calculate *V_f_*/*V_T_* and *V_r_*/*V_T_* and, so, the porosity volume fraction, *V_v_*/*V_T_*.

Small samples are also used for observation through optical microscopy; it is possible to measure the total space occupied by the pores and, by knowing the lens magnification, to infer the porosity distribution percentage. The sample surface requires previous metallurgical polishing.

For both methods, nothing can be said in terms of accuracy and reliability, since the real void content is unknown. However, in consideration of the errors involved in the measurements and owing to data present in the literature [6], it can be inferred that the void fraction obtained with the volumetric method (Archimedes) is affected by a larger deviation in comparison with optical microscopy. Of course, to minimize the section-bias errors, at least three adjacent cross-sections must be taken. Actually, the results of both volumetric and microscopy methods are strongly related to very small portions of the laminate; this, of course, makes the results unlikely to be a depiction of the whole piece.

A more reliable method may be acid digestion, since it involves the testing of the whole part [7]. However, two considerations should be made against this method. On one side, it requires the use of dangerous acids that, in turn, involves severe safety at work measures with additional costs in terms of personnel training and of specific protection devices. On the other side, the obtained porosity value can be assumed to be representative of a manufacturing process, but it cannot account for the real amount of porosity within each final part. Conversely, mainly in the aeronautical field, there is the need to assess the porosity percentage on each part of the entire production; this is the reason for the ever increasing interest on the development of non-destructive testing (NDT) methods.

### 2.2. Non-Destructive Methods

Brit and Smith [8], in a review on non-destructive evaluation (NDE) methods for porosity assessment in fiber reinforced polymers, stated that none of the current NDE techniques can be considered as a reference technique, due to the dependence of the instrument response on the pore morphology, as well as on the intrinsic nature of the fiber and matrix themselves.

Nevertheless, ultrasonic testing (UT) [9] is actually the most commonly used technique, especially in the industrial environment, in which the production processes are well consolidated, as in the aerospace sector. However, the detection of porosity in resin-starved areas of polymer matrix composites is very difficult and generally not reliable with the ultrasonic method, and so, the attention has been given also towards other methods, such as computed tomography (CT) [10] and infrared thermography (IRT) [11]. CT [10] may be used to overcome some of the UT limitations regarding size, shape and position of all individual pores. Adversely, it involves difficulties in testing real parts (it is mainly suitable for small samples), as well as safety at work concerns. Industrial production is demanding for proper inline control.

A methodology that acts in a non-destructive and completely non-invasive way and that does not involve any specific surface prerequisites, nor safety at work concerns, is flash thermography (FT). It has proven its suitability, through the evaluation of porosity percentages linked to measurements of thermal diffusivity [11]. The attention of the present work is to trace the actual state-of-the-art on the use of FT and to illustrate its suitability in comparison to the most widely used UT.

#### 2.2.1. Ultrasonic Testing

Porosity can be determined with UT by creating ecographic images (c-scans), through correlation with either a single ultrasonic frequency (narrowband approach) or the ultrasonic frequency slope (broadband approach) [12].

Amongst the empirical techniques developed, the measure of the variation of the signal attenuation is massively used, since it accounts for the scattering effect induced by voids [13]. The decrease of the intensity, *I*, of the ultrasonic signal [14] while propagating within the path length, *z*, of an attenuating medium, is well described by the classical Beer–Lambert law:

$$I(z)=I_{0}e^{-\beta z}$$ (3)

with β being the attenuation coefficient, which, providing an estimation of the bulk attenuation, is a good marker of porosity.

Further, the correlation between the signal attenuation and the amount of porosity has been demonstrated [14], as well as the dependency of the attenuation on the ultrasonic wave frequency [15]. In particular, Hsu [16] used the through-transmission (TT) technique to assess the amount of porosity as volume percentage in CFRP, and successively, Steiner [17] demonstrated that the ultrasonic pulse-echo (PE) technique is effective, too. More recently, researchers at the Italian Aerospace Research Centre (CIRA) [5] demonstrated, via PE, that the amount of porosity that forms during the curing cycle depends also on the fibers’ orientation.

The ecographic approach is very effective in the presence of spherical pores that have a diameter exceeding the instrument resolution and that are homogeneously disseminated within an isotropic medium; conversely, a quantitative determination of porosity is very difficult or not reliable. Recently, a correlation model to account for the random presence of voids in an anisotropic medium was proposed [18]. However, at the current state-of-the-art, a quantitative assessment of porosity in rather complex composite structures is not feasible.

#### 2.2.2. Flash Thermography

Flash thermography can be used to evaluate porosity from measurements of thermal diffusivity.

It derives from the flash method, which was proposed by Parker in 1961 [19] and which became an American Society for Testing and Materials (ASTM) standard in 1992 [20]. Parker’s method is based on the analytical solution of the Fourier equation with adiabatic boundary conditions for an infinite slab subjected to a thermal Dirac pulse [21]. The test is performed according to the transmission scheme, with an energy pulse being deposited on the sample front surface, while the temperature evolution is measured on the rear surface. Thermal diffusivity is linked to the sample thickness, *L*, and to the half-maximum rise time through the equation [19]:

$$\alpha =\frac{0.139L^{2}}{t_{1/2}}$$ (4)

**Figure 1.**
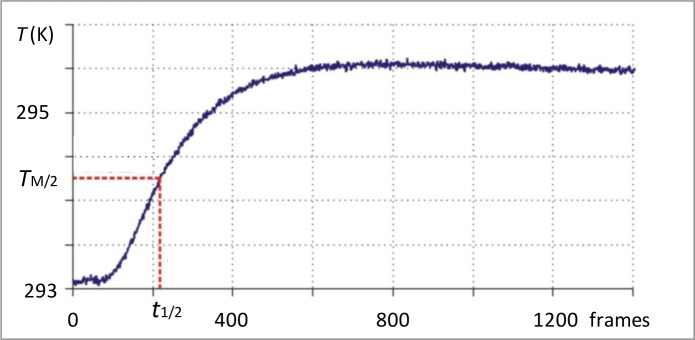
Typical temperature (*T*) plot against the number of images (frames) in a point on the specimen backside. The dotted red line shows the link between *t*_1/2_ and *T*_M/2_.

*t*_1/2_ is the time in seconds needed to reach half of the maximum temperature value, *T*_M/2_. A typical temperature (*T*) plot against the number of images (frames) is shown in Figure 1; from the frame rate, it is easy to revert to time in seconds. Of course, the frame rate is chosen according to the infrared camera performance used and the characteristics of the coupon under measure.

Thermal diffusivity is defined as:

$$\alpha =\frac{k}{\rho c_{p}}$$ (5)

where *k* is the thermal conductivity, ρ is the density and *c_p_* is the specific heat at constant pressure. Thermal diffusivity, being dependent on density and specific heat, is a good parameter for the indirect estimation of the amount of embedded porosity within the material.

Of course, this method was first conceived [19] for temperature measurements with contact transducers (e.g., leaf thermocouples); while now, it is being used in combination with infrared thermography. The usefulness of the infrared camera is obvious, since it allows for fast monitoring, in a remote way, of temperature variations and for the evaluation of the thermal diffusivity pixel-by-pixel over a given, ever large, area. The possibility to map the thermal diffusivity is an advantage in view of accounting for local variations due to the presence of local material inhomogeneities.

Some Historical Hints into the Use of Flash Thermography

An early attempt to monitor porosity in thermoplastic composites was done by Steiner *et al*. [22] through the use of thermography and laser-based ultrasonics, but much interest was raised starting from 2000. In particular, in 2001, Chu *et al.* [23], through finite element analysis (FEA) and experimental tests, demonstrated that there is a good correlation between thermal diffusivity and porosity in composite materials. A series of finite element models were built, and thermal responses for isotropic and orthotropic materials with various thermal diffusivities subjected to different heating conditions were investigated. Experimental tests are useful to validate the numerical models and to estimate the unknown parameters, like the amount of heat flux. They also show that both laser and flash heating can be used to effectively obtain thermal diffusivity.

Later, Grinzato *et al.* [24] carried out tests, with flash thermography, on a real aeronautic CFRP part to evaluate both thermal diffusivity and thermal effusivity. The latter were compared to results obtained with ultrasonics c-scans, and it was found that thermal effusivity is much more sensitive than thermal diffusivity to porosity variations, but it is also more affected by heating non-uniformities. One of the main problems encountered when testing complex structures is to differentiate between porosity (micro-voids) and resin-starved areas. It has been observed that a high porosity level decreases the thermal diffusivity much more than it does an area rich in resin; but for a better discrimination, good data filtration through mathematical procedures is necessary [25]. An investigation, aimed at discovering porosity in a real aircraft part [26], raised the problem of non-uniform heating within pulse thermography; the approach of the source distribution image (SDI) was proposed as a reference for discriminating between sound and defective zones.

A linear relation between diffusivity and porosity was experimentally obtained through flash thermography by Hendorfer *et al.* [11]. Measurements were performed on CFRP samples 2 mm-thick with simulated porosity between 1% and 5%; the average nominal porosity being estimated through ultrasonic testing. Flash thermography offers some advantages over ultrasonics in terms of detailed information supplied within a reduced testing time. Mayr and Hendorfer [27] investigated also the possibility to apply flash thermography in reflection mode and found a higher data spread with respect to the use in the transmission mode. In particular, they observed a decrease in sensitivity for both modes (transmission and reflection) when porosity increased up to 10%; this was ascribed to the tendency of pores to interlink, as verified through measurements with computed tomography. Later, Mayr *et al.* [28] carried out tests on CFRP involving prepregs woven in a twill weave pattern at a ratio of 3/1 and found active thermography comparable to ultrasonic testing in terms of the ability to determine porosity. As a main finding, they observed, through a comparison with CT data, that the thermal diffusivity is strongly affected, not only by the porosity content, but also by the pore shape.

Most recently, flash thermography proved suitable for porosity measurements also in flax/epoxy composites [29].

The usefulness of flash thermography within the evaluation of porosity in composites seems extensively demonstrated. However, many papers deal with qualitative estimation of porosity, and some refer to composites of given characteristics, not always completely specified, which make a general data correlation difficult. On the other side, before a full acceptance of a method, lots of tests are needed, as well as comparison of data coming from different laboratories worldwide and carried out on similar test articles. The latter point is very difficult to achieve, due to the variability associated with composites. In fact, composites are made of two basic ingredients: the matrix and the fibers; but each of them has different thermal properties, and then, it is their mutual percentage that drives the thermal diffusivity of the final part [28]. For example, in carbon/epoxy, carbon fibers have a thermal diffusivity much higher than the resin epoxy; then, the thermal diffusivity of a CFRP part strongly depends on the volume of carbon fibers. Wrobel *et al.* [30], through flash thermography measurements, found a variation of thermal diffusivity in the range 1.5–2.2 × 10^−7^ m^2^/s for a variation of fiber content between 13% and 28%. The fibers’ orientation also plays a fundamental role, since, as stated in [31], the direction of the heat flow coincides with the direction of the fibers. In addition, the manufacturing process involves many parameters, making it difficult and almost impossible to replicate a product of completely equal characteristics.

A more extensive investigation, involving a large number of CFRP samples of several different stacking sequences and with different percentages of porosity, was recently carried out by Toscano *et al.* [32,33]. Some samples similar to those used by Toscano *et al.* [32–34] are herein considered to better investigate the influence of the fibers’ orientation coupled with a variation of porosity percentage. Such specimens are tested with both flash thermography and ultrasonics with the purpose of a data comparison.

## 3. Experimental Section

In this section, several specimens of two stacking sequences, which were fabricated following the procedure already used by Toscano *et al.* [32–34], are considered. Data obtained with flash thermography are compared to those obtained with destructive methods and with ultrasonic testing. The intention is to check the ability of flash thermography to discriminate differences of porosity induced by a different orientation of fibers and to get quantitative data that may serve as a basis for a full assessment of flash thermography as an alternative to ultrasonics and in view of its introduction for in-line post-production inspection of composite parts.

### 3.1. Description of Specimens

Several coupons, involving a polymer (resin epoxy) as a matrix and carbon fibers as a reinforcement, were manufactured that enclosed a certain percentage of porosity and a slag inclusion [32]. More specifically, CFRP specimens include prepreg laminas M21/IM7, provided by Hexcel^®^, which were superimposed (hand lay-up technique) following two stacking sequences (Table 1). More specifically, a unidirectional and a symmetrical orientation of fibers were considered with coupons named Pu and Ps respectively. Groups of them were separately cured in an autoclave at a different pressure percentage, *P_c_*, being, respectively, *P_c_* = 100%, 75%, 50%, 25% and 0% of the prescribed one (7 bar gauge) to induce the formation of a different percentage of porosity.

**Figure 2.**
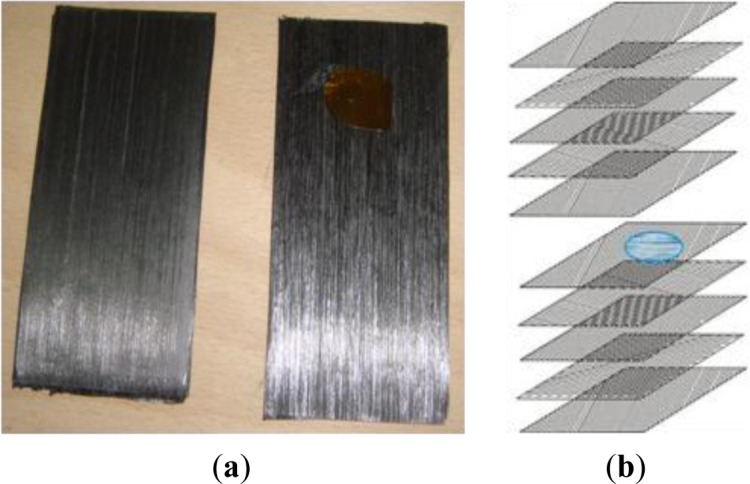
Prepreg laminas and the position of the Kapton disk. The photo on the (**a)** shows the position of the Kapton disk over unidirectional fibers. The sketch on the **(b)** shows the position of the insert on one half of the lamina in the middle of the stacking sequence.

Each specimen is 10 cm-long and 5 cm-wide and has an overall thickness of about 5 mm. A Kapton disk 20 mm in diameter and 0.06 mm thick was inserted on one half (Figure 2a) in the middle of the stacking sequence (Figure 2b) in order to account also for the presence of local delamination. The photo in Figure 2a shows the position of the Kapton disk over unidirectional fibers; such laminas are overlapped, as the sketch on the left (Figure 2b) shows. More specifically, long fiber laminas are simply overlapped to obtain Pu-type specimens or overlapped after they were cut and rotated by +45°, −45° to obtain Ps type specimens. Indeed, a slag insert acts as a zone of lower thermal diffusivity, which may serve also as a reference for quantitative thermal diffusivity measurements. Three specimens for each stacking sequence and curing pressure were manufactured, resulting in a total of 30 specimens.

### 3.2. Porosity Assessment by Destructive Methods

First of all, an estimation of the porosity percentage was performed in a destructive way by measuring the coupons’ average density. This was done by weighting in water and in air [3] small pieces that were extracted from each coupon type. Then, through the law of mixtures (properly modified introducing the volume occupied by the voids) [5], the volume fraction of the voids was obtained, *V_V_*%.

**Figure 3.**
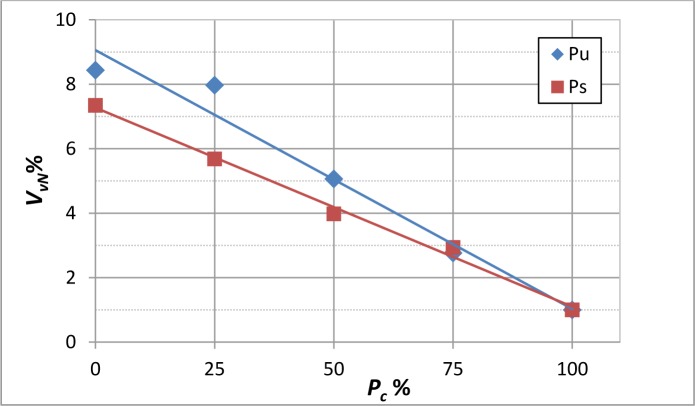
Normalized volume fraction of voids, *V_V_*%, *versus* the curing pressure, *P_c_*%, for Pu and Ps specimens.

Values of *V_V_*%, normalized with respect to the value obtained when *P_c_* = 100%, are plotted against *P_c_* in Figure 3 for specimens Pu and Ps. These specimens were chosen to be, respectively, representative of the variation of the stacking sequence from a unidirectional direction of fibers to an oblique one. As expected, for both specimen types, the percentage of porosity introduced depends almost linearly on the curing pressure following the equation:

y=a+bx (6)

with coefficients collected in Table 2.

In particular, the higher the applied pressure, the lower the amount of porosity induced in the coupons. The largest data spread lies within ±2% in the Pu-type coupons.

### 3.3. Measurements with UT

PE in immersion was chosen to perform ultrasonic testing. To prevent water infiltration, each coupon was sealed around the edge with a silicon layer. The inspection of all coupons was carried out using a flat 4–20 MHz probe (by Panametrics^®^) with a diameter of 0.25 inches (6.35 mm), tuned at 5 MHz and with a spatial resolution of 0.5 mm. The ultrasonic set-up includes a pulser-receiver unit (by Nukem^®^) and an automated system that allows the probe movements to be synchronized with the signal acquisition. The output of the inspection consists of the attenuation coefficient, β (decibels), and of the time-of-flight (ToF) (microseconds) c-scans.

**Figure 4.**
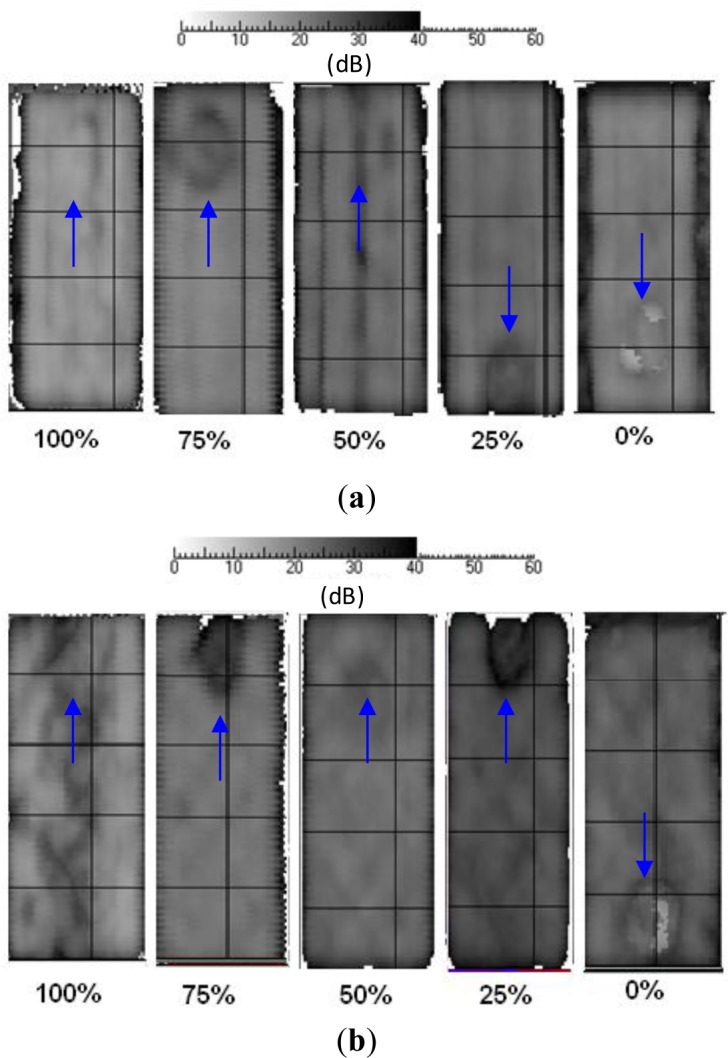
Attenuation c-scans for different *P_c_* values. (**a**) Pu coupons; (**b**) Ps coupons. An arrow indicates the position of the Kapton disk in each map.

**Figure 5.**
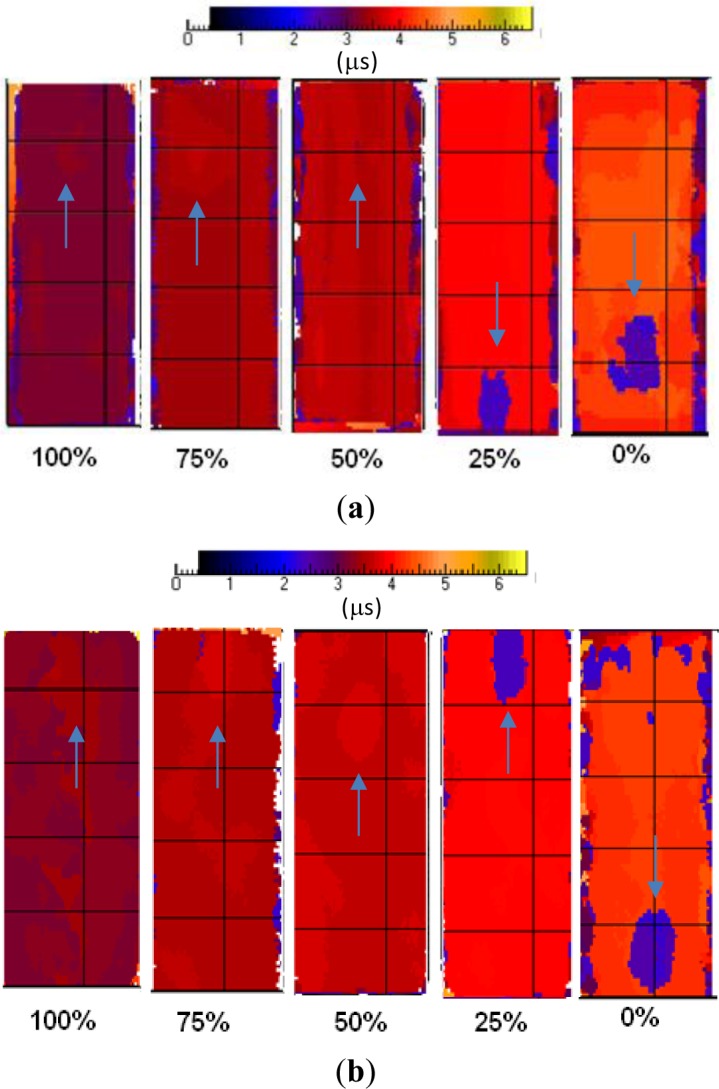
Time-of-flight (ToF) c-scan for different *P_c_* values. (**a**) Pu coupons; (**b**) Ps coupons. An arrow indicates the position of the Kapton disk in each map.

As already pointed out, β is a good marker of porosity. Therefore, from each attenuation c-scan, the average attenuation β value was measured over an area of almost 20,000 points [5,34] (excluding the area within the Kapton insert). For both coupon types, results are presented in terms of attenuation c-scans (with decibel units) in Figure 4a,b and of ToF c-scans (with microsecond units) in Figure 5a,b; an arrow indicates the position of the Kapton disk in each map. The local variations in the attenuation c-scans, principally due to the different amount of porosity in the coupons, can be appreciated by the different grey levels (Figure 4). In particular, it is easy to notice that coupons cured with *P_c_* = 0% are the darkest ones, because the massive presence of porosity strongly reduces the signal amplitude. To quantify these observations, total average attenuation values are collected in Table 3.

As can be seen, both attenuation and ToF c-scans are not always effective in outlining the Kapton disk. However, it seems that the probability of detection increases with increasing the amount of porosity in the material; or better, when the curing pressure is much less than the prescribed one. This is probably due to the fact that the Kapton disk, being very thin (*s* = 0.06 mm), does not affect the passage of the ultrasonic signal in a well-consolidated material.

On the contrary, when the amount of porosity increases (*V_vN_*_%_ > 2), a disbonding between the Kapton disk and the surrounding prepreg laminas is promoted, involving the presence of a gas layer that affects the passage of the ultrasonic signal. In this case, the Kapton disk becomes visible in the ToF c-scan because it affects the travel time of the direct reflection.

**Figure 6.**
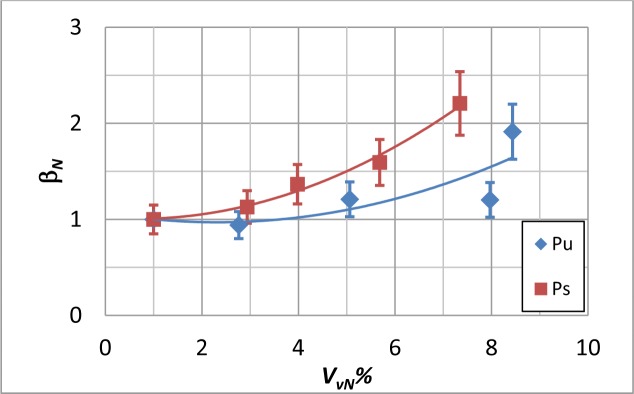
Normalized β*_N_* values against *V_VN_*% for Pu and Ps coupons.

At last, to better account for the fibers’ orientation, the average β value was normalized with respect to the value obtained when *P_c_* = 100%; the so obtained values, which are named β*_N_* are plotted against *V_vN_*% in Figure 6. As can be seen, the Ps coupons display a larger variation of the β*_N_* value; instead, the Pu specimens show a lower variation of β*_N_*, but a larger data spread. For Ps and Pu coupons, data are fitted by a polynomial regression:

$$y=a+bx+cx^{2}$$ (7)

with correlation coefficients collected in Table 4. The plots in Figure 6 display also the error bars to account for the variability of data with respect to the correlation curve. For both coupon types, the data spread increases with increasing the void percentage.

### 3.4. Measurements with Flash Thermography

A Hensel^®^ flash head and power supply, able to emit 6000 Joules in 1/400 s, was used to instantaneously heat up the inspected specimen [32]. The temperature-time variation on the opposite side of the specimen was monitored by the infrared camera, SC5000 (Flir Systems, Paris, France). This camera is equipped with 320 × 256 InSb focal plane array Stirling cooled detector working in the 3.7–5.1 μm infrared wavelength band and of a thermal resolution of 25 mK. The spatial resolution, in the present tests, was 3 pixels/mm, and the frame rate was 5 Hz.

**Figure 7.**
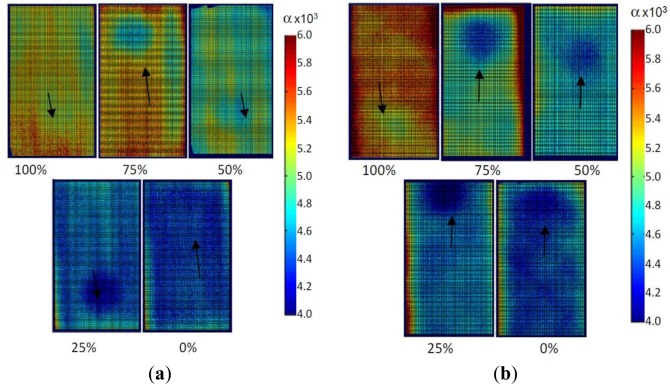
Thermal diffusivity maps. (**a**) Pu-type coupons; (**b**) Ps-type coupons. An arrow indicates the position of the Kapton disk in each map.

Through the analysis of the image sequence with an *ad hoc* developed MATLAB tool and by applying Equation (4), thermal diffusivity maps were obtained. They display thermal diffusivity values that were evaluated point-by-point over the entire surface of each coupon as average values through the specimen thickness. Two thermal diffusivity maps for coupon types Pu and Ps are reported in Figure 7a,b with α expressed in 10^−3^ cm^2^/s.

**Figure 8.**
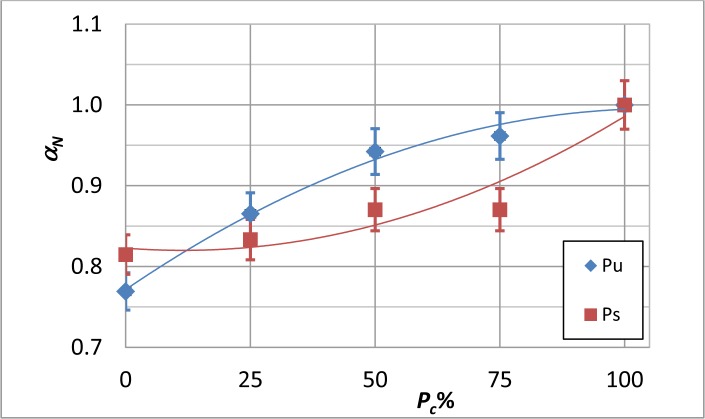
Normalized average thermal diffusivity, α*_N_*, *versus P_c_*% for both Pu and Ps coupon types.

**Figure 9.**
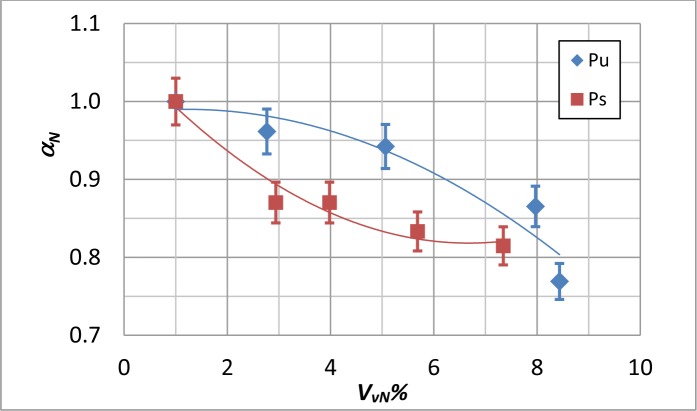
Normalized average thermal diffusivity, α*_N_*, *versus V_vN_*% for both Pu and Ps coupon type plots.

As can be immediately noticed from the variation of color, for each map, low values (see the color bar on the right) of thermal diffusivity are attained in correspondence with the location of the Kapton disk, which is visible in almost all the coupons, although in some cases, it is not clearly outlined; to facilitate readability, an arrow indicates the position of the Kapton disk. For coupons cured with *P_c_* = 0%, the relevant amount and distribution of porosity has caused a sort of blurring effect, which makes ambiguous the discrimination of the Kapton disk contour. In addition, assuming α values over the Kapton disk as the lowest ones, it is possible to clearly see, as the distribution of the thermal diffusivity is driven by the fibers’ orientation. In particular, a pack-threads effect of fibers is observed mainly for Pu specimens cured at *P_c_* = 50% and 25%. On the other side, this effect was already visualized [34] within unidirectional fibers as mainly pronounced for the shorter fibers oriented at 90° (along the shorter side). For the plots in Figures 8 and 9, the error bars are visualized to account for the variability of data with respect to the correlation curve. On average, the larger data spread is attained by the Pu coupon type.

For each coupon, an average thermal diffusivity value was calculated over the sound material (excluding the area containing the Kapton disk); such values were then normalized with respect to the thermal diffusivity value of the coupon with *P_c_* = 100%. The so obtained values, which are named α*_N_*, are plotted against *P_c_* and against the normalized volume of voids *V_vN_*%, respectively, in Figure 8 and in Figure 9.

From Figures 7 and 9, it is possible to notice that:

- the thermal diffusivity tends to decrease with decreasing *P_c_* (*i.e.*, increased embedded porosity);
- the largest reduction with decreasing *P_c_* of about 23% is registered for the coupon type, Pu;
- the average value, as well as the distribution of the thermal diffusivity is affected by the fibers’ direction.

It is known that thermal diffusivity depends on the fibers’ direction [31,35]. The curing pressure plays a fundamental role in the formation of voids, but this pressure-dependent effect is also found to be dependent on the stacking sequence. Probably, gases can, under pressure, more easily escape from a unidirectional laminate, than from a more complex stacking sequence in which they may remain entrapped within the labyrinth of fibers; this may explain the larger increase of the thermal diffusivity with increasing the curing pressure for specimens with unidirectional fibers (Figure 8).

Data in both Figures 8 and 9 are well fitted by a second-order polynomial, like Equation (7), with coefficients collected in Table 5 for the α*_N_* to *P_c_* correlation (Figure 8) and in Table 6 for the α*_N_* to *V_vN_* correlation (Figure 9). As a main finding, a change of slope is observed for the two different specimen types. This feature, which may appear almost strange at first sight, is surely to be ascribed to the fibers’ orientation and will be better discussed in the next section.

### 3.5. Comparison between Thermographic and Ultrasonic Data

**Figure 10.**
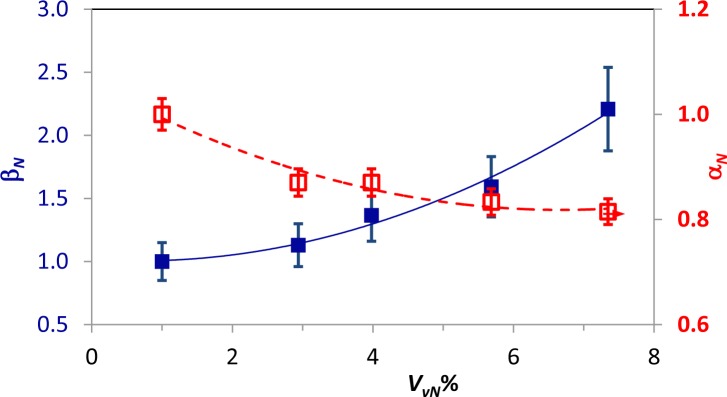
Comparison between ultrasonic attenuation and thermal diffusivity for the Ps coupons type. The results related to ultrasonic attenuation, β*_N_*, are in blue with the scale on the left axis, and the ones related to thermal diffusivity, α*_N_*, are in red with the scale on the right axis.

**Figure 11.**
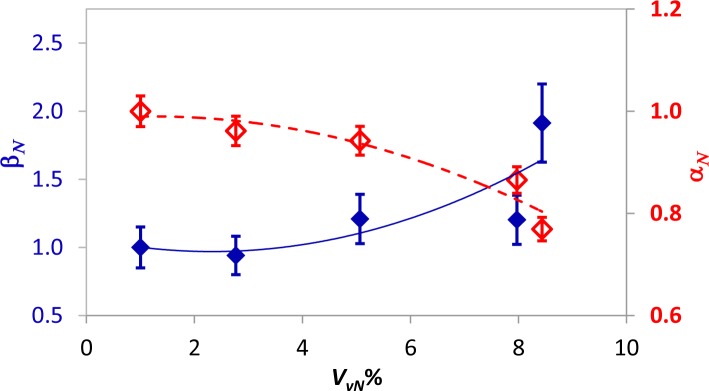
Comparison between ultrasonic attenuation and thermal diffusivity for the Pu coupons type. The results related to ultrasonic attenuation, β*_N_*, are in blue with the scale on the left axis, and the ones related to thermal diffusivity, α*_N_*, are in red with the scale on the right axis.

For a comparison between FT and UT, α*_N_* and β*_N_* are plotted together against *V_vN_*% in Figures 10 and 11, respectively, for the Ps and the Pu coupon types. As already shown, the functional relation can be fitted, for every dataset, by a second-order polynomial [Equation (7)] in a least squares sense. Thermal diffusivity decreases, while the ultrasonic attenuation increases with increasing the porosity content; β*_N_* displays larger variations with *V_vN_*%, but it displays also a larger data spread, as the error bars show. This observation is in general agreement with the literature [28].

Within the data comparison, it is also worth noting that for each specimen, due to the manufacturing process, one surface is perfectly smooth, while the other one is almost rough. In general, the rough side is characterized by a higher emissivity, making it the preferred one to be viewed by the infrared camera for tests with FT. Indeed, some specimens were tested twice by considering one time the smooth side and, after, the rough one, or *vice versa*; on the whole, no differences were found. On the contrary, ultrasonic tests were carried out on the smooth side, since, as is well known, the surface finishing is of great concern for UT.

Thus, due to the many advantages offered and in agreement with previous literature, amongst others’ reference [28], flash thermography can be preferred to ultrasonic for porosity assessment in composites.

Going more in depth into a comparison of Figure 10 to Figure 11, a change of slope in the curve fitting α*_N_* data for both specimen types can be noted. More specifically, the curve fitting β*_N_* (ultrasonic) data displays always, for both specimen types, Pu and Ps, the same concavity, which is also in agreement with [28]. Conversely, a change of concavity is observed for α*_N_* data by changing the type of specimen. This effect is certainly to be ascribed to the different type of material in the sense that the distribution and orientation of fibers, as well as their mutual position play a fundamental role in porosity formation. Mayr *et al.* [28] already observed an abrupt drop of thermal diffusivity at a porosity of about 0.5%; they ascribed such a non-linear correlation between thermal diffusivity and porosity to the variation of the pore shape. In particular, they stated that the pores tend to flatten with increasing the porosity percentage and have a degradation effect on the heat flux, decreasing, in turn, the thermal diffusivity. From Figure 10, which refers to the Ps specimen type, there is first an abrupt drop of the thermal diffusivity with increasing porosity, followed by a much milder decrease; the contrary happens for specimen type Pu in Figure 11. Of course, the space allowing pores to assume either a spherical or a flattened shape is different for fibers aligned in a unidirectional way (Pu) or fastened in a cross-fashion (Ps). Unfortunately, there is no possibility to compare data with the literature, the materials used being different; in particular, the material used in [28] is a plain weave one. Even if the behavior of the Ps type may be assumed to be similar to that of the plain weave for certain porosity percentages, which may explain the initial abrupt drop of α*_N_* with *V_vN_*%, the change of slope with the stacking sequence deserves further attention.

## 4. Conclusions

The use of flash thermography to assess the amount and distribution of porosity in composites has been investigated. In particular, the most relevant literature has been reviewed, and some new experimental data have been shown to complete the actual state-of-the-art in the use of flash thermography.

The new data come from a set of specimens, including a different amount of porosity, two different stacking sequences and, also, slag inserts, to simulate local delamination. The results obtained with flash thermography were compared with those coming from ultrasonic testing; the percentage of porosity was determined by the destructive volumetric method. A comparison with data present in the literature was attempted, but it was not at all possible, the involved materials being different.

To sum up, it has been demonstrated, in agreement with the literature, that the measure of thermal diffusivity by flash thermography, as a parameter for porosity evaluation, is alternative to the typical ultrasonic attenuation estimation, which is commonly used, especially, in the aeronautical field. However, flash thermography offers some advantages, since it is effective, non-contact (no coupling media are necessary), fast and is also not affected by the surface finishing, meaning that, unlike UT, a part can be inspected viewing the smooth or the rough side indifferently. In addition, flash thermography allows one to contemporaneously detect manufacturing defects and assess the porosity amount within only one test with, of course, economic advantages. The test setup can be easily incorporated in the industrial enterprise for the in-line inspection of parts. In addition, as an important remark, non-destructive testing with IRT is carried out using a simple and safe (for the personnel) set-up arrangement without any further issues in terms of safety at work concerns.

As a final point, an important outcome regards the influence of the stacking sequence on the correlation between thermal diffusivity and porosity. This feature deserves further attention for a full assessment of the validity of flash thermography.

## Figures and Tables

**Table 1.** Specimen characteristics.

Coupon Type	Stacking Sequence	No. of Plies
Pu	[0°]	24
Ps	[45°/−45°]_s_	24

**Table 2.** Coefficients of Equation (6) for plots shown in Figure 3.

Coupon Type	*a*	*b*	*R*^2^
Pu	9.061	0.080	0.968
Ps	7.276	0.062	0.994

**Table 3.** Attenuation values and standard deviation for both (Pu and Ps) types of specimens.

*P_c_*%	Pu Total Attenuation (dB)	SD	Ps Total Attenuation (dB)	SD
100	22.5	1.2	20.7	3.4
75	22.1	0.9	22.5	0.7
50	25.0	2.2	25.6	0.9
25	25.6	1.1	29.1	1.1
0	33.5	1.7	38.3	1.2

**Table 4.** Coefficients of Equation (7) for β*_N_* to *Vv_N_* correlation (Figure 6).

Coupon Type	*a*	*b*	*c*	*R*^2^
**Pu**	1.067	−0.084	0.018	0.665
**Ps**	1.016	−0.034	0.026	0.987

**Table 5.** Coefficients of Equation (7) for α*_N_* to *P_c_* correlation (Figure 8).

Coupon Type	*a*	*b*	*c*	*R*^2^
Pu	0.771	0.0042	−2 × 10^−5^	0.989
Ps	0.823	−0.0005	2 × 10^−5^	0.907

**Table 6.** Coefficients of Equation (7) for α*_N_* to *V_vN_* correlation (Figure 9).

Coupon Type	*a*	*b*	*c*	*R*^2^
Pu	0.984	0.009	−0.004	0.905
Ps	1.059	−0.072	0.005	0.957
